# Genetic Diversity and Nodulation Potential of *Bradyrhizobium* Strains in Cowpea and Soybean

**DOI:** 10.3390/plants14243857

**Published:** 2025-12-18

**Authors:** Camila Pereira de Moraes Carvalho, Alberto Fernandes Oliveira, Luc Felicianus Marie Rouws, Fernanda dos Santos Dourado, Marcia Reed Rodrigues Coelho, Bruno José Rodrigues Alves, Jerri Édson Zilli

**Affiliations:** 1 Instituto de Agronomia, Universidade Federal Rural do Rio de Janeiro, BR 465, km 07, Seropédica 23897-035, RJ, Brazil; camilammoraes.cm@gmail.com; 2 Embrapa Agrobiologia, BR 465, km 07, Seropédica 23891-000, RJ, Brazil; afojunior@gmail.com (A.F.O.J.); marcia.coelho@embrapa.br (M.R.R.C.); bruno.alves@embrapa.br (B.J.R.A.)

**Keywords:** biological nitrogen fixation, *Vigna unguiculata*, *Glycine max*, rhizobium, tropical soil

## Abstract

*Bradyrhizobium* is a genetically diverse genus that forms symbioses with numerous legumes, including major crops such as cowpea (*Vigna unguiculata*) and soybean (*Glycine max*). Understanding the genetic and symbiotic diversity of native strains is essential for improving inoculant technologies and enhancing biological nitrogen fixation in tropical agricultural systems. This study investigated *Bradyrhizobium* strains associated with these two legumes grown in adjacent tropical soils in Brazil to elucidate their genetic relationships, taxonomic placement, and host compatibility. A total of 34 *Bradyrhizobium* strains isolated from cowpea and soybean nodules were characterized using multilocus phylogenetic analyses (16S rRNA, *gyrB*, *recA*, and *nodC*). Selected strains underwent whole-genome sequencing for comparative analyses based on average nucleotide identity (ANI) and digital DNA–DNA hybridization (dDDH). Cross-inoculation assays were performed to evaluate nodulation capacity and symbiotic efficiency on both hosts. The strains displayed high genetic diversity, forming multiple phylogenetic clusters. Most grouped within the *B. elkanii* superclade, whereas several occupied divergent lineages, some potentially representing new taxa. Genome-based analyses supported these findings, showing intracluster ANI values above 95–96% and intercluster values below 94%. A distinct group of cowpea-derived strains exhibited high symbiotic efficiency but low genomic similarity to known type strains, suggesting the presence of a novel species with potential use in inoculants. In contrast, some soybean-derived strains were genetically identical to commercial inoculants, indicating persistence or re-isolation from previously inoculated soils. Notably, strain BR 13971, isolated from soybean, nodulated both hosts efficiently, demonstrating a broad host range and suggesting a unique symbiovar. Cross-inoculation assays showed that soybean-derived strains effectively nodulated cowpea, whereas cowpea-derived strains did not nodulate soybean, indicating asymmetrical host compatibility. Particularly for cowpea, strains BR 10926 and BR 10750 demonstrated higher symbiotic efficiency than the strains currently recommended for this crop. Overall, these findings enhance the understanding of *Bradyrhizobium* diversity in tropical soils and highlight promising native strains for future inoculant development.

## 1. Introduction

Given the growing global demand for sustainable agricultural practices and the need to reduce dependence on synthetic nitrogen fertilizers, biological nitrogen fixation (BNF) through legume–rhizobia symbioses has gained renewed attention. The strategic use of efficient rhizobial inoculants not only enhances crop productivity but also contributes to soil fertility, while mitigating greenhouse gas emissions and environmental impacts associated with industrial nitrogen inputs.

Among the rhizobia, the genus *Bradyrhizobium* comprises an ancient group of bacteria capable of establishing symbiotic nodulation with diverse subfamilies within the Fabaceae (Leguminosae) family [1,2,3,4]. These bacteria are ubiquitous in tropical soils and are among the most extensively studied rhizobia in agriculture owing to their symbiotic, nitrogen-fixing associations with economically important leguminous crops [5,6,7]. Notably, *Bradyrhizobium* exhibits the broadest host range among rhizobia, forming effective associations with legumes from at least 24 of the 33 known nodulating tribes within Fabaceae [8]. This remarkable host compatibility partially helps explain the genus’s predominance in subtropical and tropical regions characterized by high legume diversity [9].

In South America alone, soybean (*Glycine max*) cultivation depends on annual inoculation of over 40 million hectares with *Bradyrhizobium* strains [10], enabling the biological fixation of more than 8–10 million tons of nitrogen per year, taking in account an amount over 200 kg ha^−1^ for each harvest [5,11]. Brazil hosts the world’s largest commercial inoculant program, with economic savings exceeding US$ 15 billion annually in nitrogen fertilizer replacement [12]. As the world’s most important oilseed crop, soybean derives substantial benefits from symbiosis with *Bradyrhizobium*, particularly in the acidic, nutrient-poor soils typical of tropical regions [13,14]. However, native (non-agricultural) soils in Brazil often lack indigenous rhizobia capable of nodulating soybean, likely due to its high degree of host selectivity [15,16,17].

Traditionally, soybean was thought to form symbioses exclusively with certain *Bradyrhizobium* species, primarily *B. elkanii* and *B. japonicum*. However, recent data suggest that some strains from other genera, such as *Ensifer* (*Sinorhizobium*) and even *Paraburkholderia* (Betaproteobacteria class), may also be able to nodulate soybean, although *Bradyrhizobium* strains consistently exhibit superior symbiotic efficiency [16,18,19]. Additionally, *Bradyrhizobium* strains demonstrate a notable genomic plasticity, which involves horizontal gene transfer and the mobilization of symbiosis islands, which may have facilitated the adaptation of native populations in the Brazilian savannas to form effective symbioses with introduced soybean [16,19].

Cowpea (*Vigna unguiculata*), another key tropical legume, also benefits substantially from associations with *Bradyrhizobium* for nitrogen acquisition. However, unlike soybean, cowpea is highly promiscuous, forming nodules with a wide range of native rhizobia in tropical soils [20,21]. This broad symbiotic compatibility has long been recognized and led to the designation of a “cowpea miscellany”, encompassing diverse strains with wide host ranges [22,23]. While cowpea primarily associates with alpha-rhizobia, especially *Bradyrhizobium*, beta-rhizobia such as *Paraburkholderia* have also been isolated from its nodules [24].

Beyond agricultural systems, *Bradyrhizobium* is a key component of soil microbial communities, even in the absence of legume hosts. In forest ecosystems, non-symbiotic and often non-diazotrophic *Bradyrhizobium* strains contribute to organic matter decomposition and carbon cycling [25]. Phylogenomic analyses have revealed substantial diversity within the genus, with seven major clades (or “supergroups”) identified [26,27]. These include the well-characterized *B. japonicum* and *B. elkanii* supergroups, a photosynthetic clade and two newly defined nodulating groups: the *B. jicamae* clade (composed of slow-growing isolates) and the Kakadu supergroup (comprising strains from northern Australia and Central America) [26]. Interestingly, some of the most divergent lineages consist exclusively of non-symbiotic, non-diazotrophic strains from forest environments, suggesting possible evolutionary constraints on the acquisition of nodulation genes [28,29].

Despite the success of commercial inoculants, challenges remain in ensuring consistent field performance across diverse soils and climates. Strain competitiveness, ecological persistence, diversity and compatibility with native microbial communities are persistent barriers to maximizing the benefits of inoculation [30,31].

In Brazil, four *Bradyrhizobium* strains are officially recommended for soybean inoculation: *B. elkanii* SEMIA 587 and BR 29, *B. japonicum* CPAC 7, and *B. diazoefficiens* CPAC 15. Similarly, four strains are approved for cowpea: *B. yuanmingense* BR 3267, *B. pachyrhizi* BR 3262, *B. amazonense* INPA 13-11B^T^, and *B. viridifuturi* UFLA 03-84 [32,33,34,35].

In this study, we analyzed two bacterial collections comprising strains isolated from soybean and cowpea nodules from adjacent sites at the experimental field station of Embrapa Agrobiologia in the southeastern region of Brazil. Despite the contrasting microsymbiont specificities of these legumes, we evaluated the strains for their ability to induce nodules on both species, their symbiotic efficiency under controlled conditions, as well as for their genetic diversity based on 16S rRNA, *recA*, *gyrB*, and *nodC* gene sequences and complemented with whole-genome analyses. This integrative approach aims to identify cross-compatible efficient *Bradyrhizobium* strains adapted to tropical conditions, thereby contributing to the development of next-generation inoculants and the sustainable use of native microbial resources in agriculture.

## 2. Results

### 2.1. Phylogenetic Analyses

**Figure 1:**
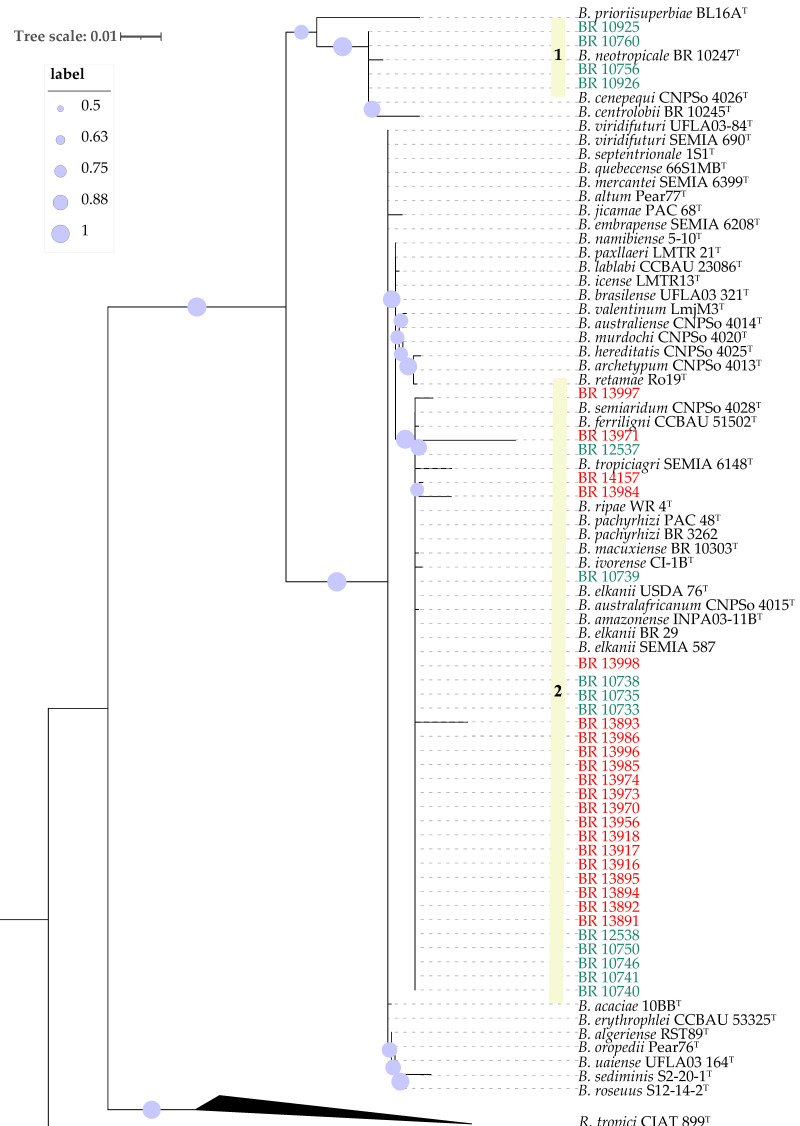
Maximum likelihood phylogeny of partial 16S rRNA sequences of *Bradyrhizobium* strains. *Rhizobium tropici* CIAT 899^T^ was used as outgroup. The strains labeled in green were isolated from cowpea and the ones in red from soybean. Bootstrap values over 50% based on 500 replicates are shown.

BLASTn comparisons of the 16S rRNA gene sequences of the 34 strains were performed using BLAST+ v2.17.0 against the NCBI nucleotide database and the EzBioCloud 16S database (version 2024), revealing that all strains were affiliated with the genus *Bradyrhizobium*. Phylogenetic analysis of 16S rRNA sequences clustered all soybean strains and ten of cowpea strains together with nine type strains of species closely related to *B. elkanii* (USDA 76^T^) as well as with the recommended soybean inoculant strains SEMIA 587 and BR 29 (*B. elkanii)*, the cowpea inoculant strains BR 3262 (*B. pachyrhizi*) and INPA 03-11B^T^ (*B. amazonense*) The cowpea inoculant strain UFLA 03-84 does not fit into this group (Figure S1; Figure 1). An exception was observed for strains BR 10925, BR 10926, BR 10756, and BR 10760, all isolated from cowpea, which grouped with *B. centrolobii* BR 10245^T^, *B. cenepequi* CNPSo 4026^T^ and *B. neotropicale* BR 10247^T^ (Figure 1).

**Figure 2:**
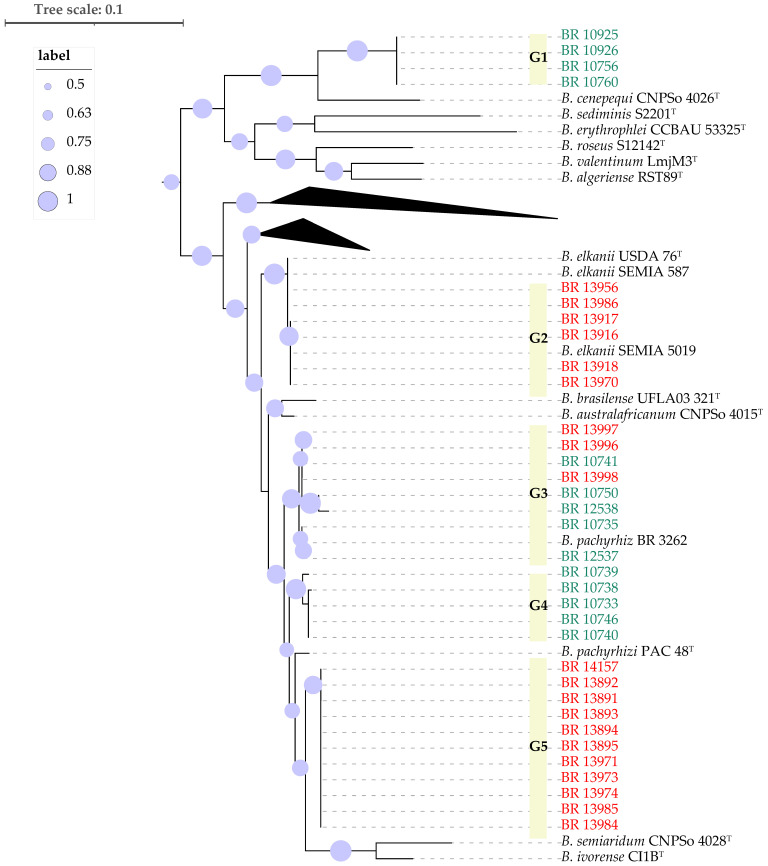
Maximum likelihood phylogeny of partial genes *recA* and *gyrB* concatenated. The strains labeled in green were isolated from cowpea and the ones in red from soybean. Bootstrap values higher than 50% based on 500 replicates are shown.

Next, partial sequences of the *recA* and *gyrB* genes (approx. 900nt) from the cowpea and soybean strains were concatenated and compared to those of the closest type strains of *Bradyrhizobium* species (Figure 2). The new strains were distributed in four well-supported groups. As in Figure 1, the cowpea strains BR 10760, BR 10756, BR 10926 and BR 10925 formed a monophyletic group (G1) that was most closely related to *B. cenepequi* CNPSo 4026^T^ (Table 1, Figure 2). A Second group (G2) consisted of six strains isolated from soybean from inoculated plots, except one (BR 13986) that was isolated from an uninoculated plant (Table 1). These soybean strains were close to the type strain of *B. elkanii* USDA 76^T^ and the soybean inoculant *B. elkanii* strains, SEMIA 587 and SEMIA 5019 (Table 1, Figure 2). The third group (G3) included five strains isolated from cowpea and three from soybean, as well as *B. pachyrhizi* strain BR 3262, which is one cowpea inoculant strain. Cowpea strains originated mainly from a pasture area, but one that was isolated from a remnant forest. The soybean strains were all from inoculated plants (Table 1). The fourth group (G4) was composed solely of five strains isolated from cowpea cultivated in forest remnants. The last group (G5) consisted of 11 highly similar strains, most of which were isolated from non-inoculated soybean plots and three from inoculated plots. This group was close to the type strains CI-1B^T^ of *B. ivorense*, and CNPSo 4028^T^ of *B. semiaridum* (Figure 2).

**Figure 3:**
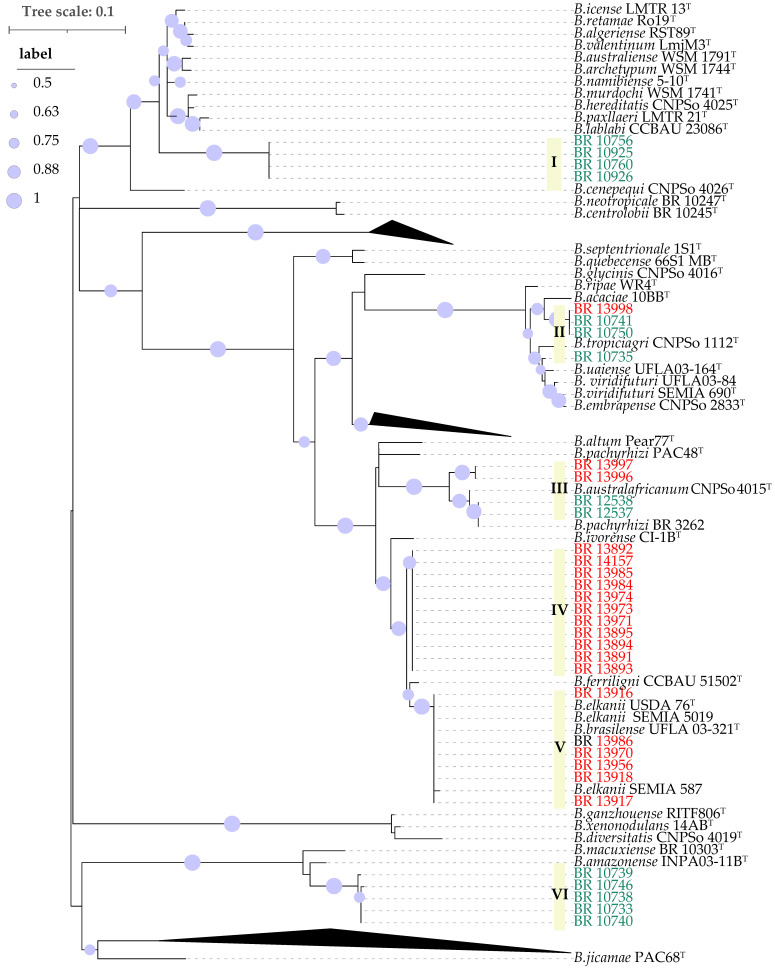
Maximum likelihood phylogeny of partial *nodC* gene sequences. The strains labeled in green were isolated from cowpea and the ones in red from soybean. Bootstrap values over 50% based on 500 replicates are shown.

The *nodC* genes analysis distributed the 34 analyzed strains across six distinct groups, rather than forming a single monophyletic group (Figure 3). The first one (I) corresponds to the same strains classified as G1 in the *gyrB-recA* phylogeny (BR 10760, BR 10756, BR 10926 and BR 10925). The *gyrB-recA* phylogeny G3 composed by cowpea and soybean derived strains (Figure 2), was separated into two clearly distinct *nodC* phylotypes, represented by groups II and III in Figure 3. Group II was most close related to *B. acaciae* 10BB^T^, *B. tropiciagri* CNPSo 1112^T^, *B. uaiense* UFLA03-164^T^, *B. viridifuturi* UFLA03-84, *B. viridifuturi* SEMIA 690^T^ and *B. embrapense* CNPSo 2833^T^, while group III showed high similarity to *B. australafricanum* CNPSo 4015^T^ and *B. pachyrhizi* PAC48^T^. Group IV joined the same strains from G5 of the *gyrB-recA* phylogeny in an unique cluster, which was also close to Group V that encompassed the same strains as G2 of the *gyrB-recA* analysis, showing highest similarity to *B. elkanii* SEMIA 587 and *B. elkanii* SEMIA 5019, as well as *B. elkanii* USDA 76^T^ and *B. brasilense* UFLA03-321^T^ and *B. ferriligni* CCBAU 51502^T^. Finally, group VI comprised five strains isolated from cowpea, corresponding to G4 of the *gyrB-recA* phylogeny with *B. amazonense* INPA03-11B^T^ as the closest reference strain (Figure 3).

### 2.2. Genome Sequencing, Average Nucleotide Identity (ANI) and Digital DNA–DNA Hybridization (dDDH)

Based on 16S rRNA, *recA-gyrB* phylogenies the strains BR 10926, BR 13971, BR 10750, BR 13996 and BR 13998 were chosen for genome sequencing, and their assembled genomes were further investigated. Details of the analysis are provided as supplementary information (Table S1). The genome assemblies showed a coverage of at least 11×, with the number of contigs ranging from 1 to 136 (Table S1). The genomic features included genome sizes between 8 and 10 Mb and a GC content of 62.5–64%.

**Figure 4:**
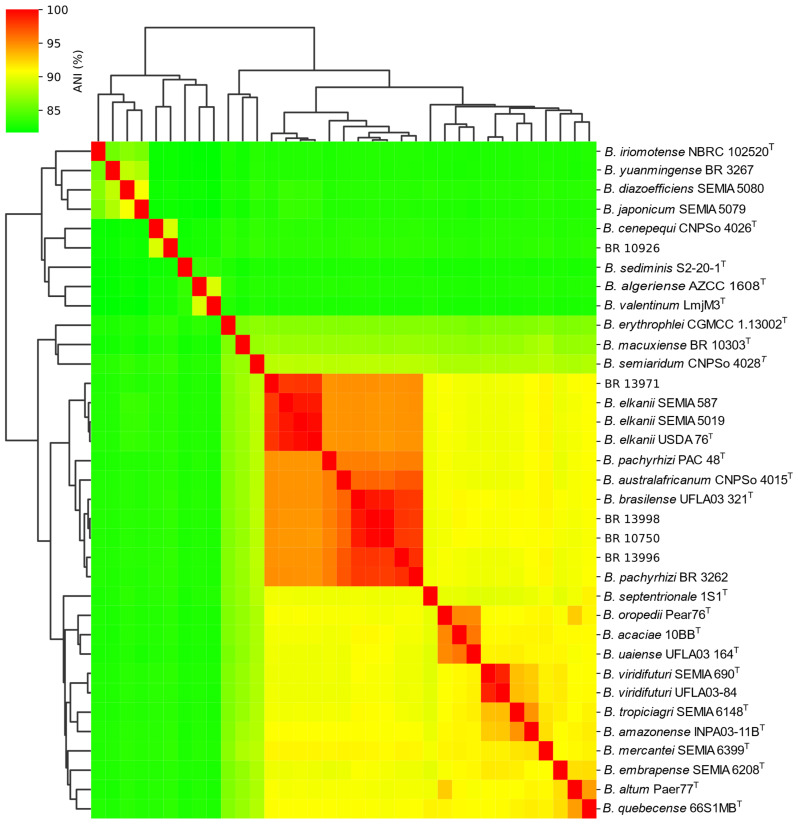
Heatmap and hierarchical clustering dendrogram based on Average Nucleotide Identity (ANI) among *Bradyrhizobium* genomes. The color gradient represents ANI values, ranging from green (lower similarity, ~85%) to red (higher similarity, up to 100%).

The ANI values among various *Bradyrhizobium* genomes, together with a hierarchical clustering dendrogram heatmap (Figure 4), demonstrated that strains BR 10750, BR 13996, and BR 13998 form a complex cluster, exhibiting values above 94% with the type strains *B. pachyrhizi* PAC 48^T^, *B. brasilense* UFLA03-321^T^, *B. australafricanum* CNPSo 4015^T^, and *B. elkanii* USDA 76^T^, as well as with the recommended strains for soybean, *B. elkanii* SEMIA 587 and *B. elkanii* 5019, and the strain recommended for cowpea, *B. pachyrhizi* BR 3262. Moreover, strains BR 10750, BR 13996, and BR 13998 showed ANI values greater than 98% among themselves (Table 2), indicating high genomic similarity. Additionally, the ANI values for these strains were also above 98% when compared with *B. brasilense* UFLA03-321^T^ (99.0%, 98.1%, and 99.2%, respectively), further reinforcing their close relationship with this species. Regarding *B. pachyrhizi* PAC 48^T^, the ANI values for BR 10750, BR 13996, and BR 13998 were 95.2%, 95.4%, and 95.4%, respectively, whereas for *B. australafricanum* CNPSo 4015^T^, the observed values were 96.2%, 96.9%, and 96.4%, respectively. Specifically, strains BR 10750, BR 13996 and BR 13998 all presented ANI values above 96.4% with *B. brasilense* UFLA03-321^T^ and *B. australafricanum* CNPSo 4015^T^. Notably, a high ANI value of 96.3% was also observed between *B. brasilense* UFLA03-321^T^ and *B. australafricanum* CNPSo 4015^T^.

On the other hand, strain BR 13971 exhibited ANI values below 95% with strains BR 13996, BR 13998, and BR 10750; however, it showed an ANI of 97.3% with *B. elkanii* USDA 76^T^, indicating that BR 13971 belongs to thatspecies. Finally, BR 10926 presented ANI values below 85% in all comparisons, and 87.86% with the closest strain, *B. cenepequi* CNPSo 4026^T^, demonstrating that it is genetically more distant from the other strains and described *Bradyrhizobium* species.

Digital DDH analysis revealed a pattern consistent with the ANI results. Strain BR 13971 exhibited a dDDH value of 83.6% with *B. elkanii* USDA 76^T^, supporting its classification within this species. For strain BR 10926, the highest dDDH value was 39.5% in comparison with *B. cenepequi* CNPSo 4026^T^, with all other comparisons showing lower values (Table 2). Similarly to the ANI results, the dDDH values among strains BR 10750, BR 13996, and BR 13998 were above 84%. All three strains also exhibited values slightly above 70% with *B. australafricanum* CNPSo 4015^T^ and *B. brasilense* UFLA03-321^T^, with the exception of strain BR 10750 which presented a 68.9% dDDH when compared to *B. brasilense* UFLA03-321^T^ (Table 2). The dDDH value between *B. australafricanum* CNPSo 4015^T^ and *B. brasilense* UFLA03-321^T^ was exactly 70% (Table 2).

### 2.3. Symbiotic Performance of Strains on the Two Host Plants

In the first experiment, all strains isolated from soybean were able to nodulate both their original host and cowpea (Table 1). In contrast, strains isolated from cowpea were able to nodulate only this host, despite belonging to common phylogenetic groups, such as G3 from the *gyrB-recA* phylogeny (Figure 2); this group is equivalent to group II and III from the *nodC* phylogeny (Table 1). There was considerable variation among strains in their ability to form nodules and promote biomass accumulation, with soybean-derived strains generally exhibiting higher efficiency for this crop (Table 1). The strains that stood out most for soybean across all three variables were BR 13956 and BR 13918. Conversely, in cowpea, the most efficient strains were not necessarily those originally isolated from this host (Table 1). Strain BR 13971, isolated from soybean, promoted the highest biomass production, followed by strains BR 10750 and BR 10926, both isolated from cowpea. However, BR 10750 and BR 10926 were among the strains that induced the lowest nodule number and mass, being the strain BR 13971 an exception, as it promoted greater biomass, as well as higher nodule number and mass (Table 1).

In the second experiment, the symbiotic efficiency of four strains that showed promising results in the first experiment with cowpea were evaluated in comparison to strain BR 3262, which is recommended for this crop (Table 3). Similar to BR 3262, strains BR 10926 and BR 10750 induced more than 100 nodules per plant, while strain BR 13956 resulted in a significantly lower nodule number (Table 3). Regarding nodule dry mass, inoculation with strains BR 10926 and BR 10750 led to values approximately three times higher than those obtained with the recommended strain (83.7 and over 200 mg per plant). A similar trend was observed for shoot dry mass, with BR 10926 and BR 10750 outperforming the recommended strain significantly and presenting values comparable to the nitrogen control (Table 3). Strain BR 13971, isolated from soybean, also performed similarly to BR 10926 and BR 10750, which was not the case for BR 13956 (Table 3).

In the third experiment, conducted with soybean, strain BR 13956 presented similar results in all three categories evaluated when compared with strain SEMIA 5079, recommended for soybean, while strain BR 13971 presented lower values for nodule dry mass and plant dry mass (Table 3). For nodule dry mass, only plants inoculated with strain BR 13956 exhibited values equivalent to the recommended strain. On the other hand, shoot dry mass in plants inoculated with BR 13956 was similar to both the recommended strain (4,3 g per plant) and the nitrogen control, and significantly higher than the other treatments, including the uninoculated control (Table 3).

## 3. Discussion

The strains evaluated in this study were isolated from cowpea and soybean plants cultivated in soils from adjacent areas (Table 1). In greenhouse cross-inoculation experiments, the strains were tested on both hosts, revealing distinct nodulation patterns (Table 1). While soybean-derived strains were able to nodulate both soybean and cowpea, cowpea-derived strains formed nodules exclusively on this host. This observation supports the well-established concept that cowpea has a broad host range and it is capable of nodulating with a wide variety of rhizobial strains from diverse phylogenetic backgrounds [20,23,36], whereas soybean establishes a more specific symbiotic interaction, forming nodules with a limited number of compatible strains [16,37]. In this context, cowpea appears to possess a greater capacity to recruit phylogenetically diverse symbiotic partners than soybean [22,23]. Furthermore, certain strains originally isolated from soybean demonstrated higher nodulation efficiency, as indicated by increased biomass accumulation in both soybean and cowpea (Table 1).

Phylogenetic analysis based on 16S rRNA sequences revealed that the 34 strains clustered into two major groups, with 30 of them closely related to the so-called *B. elkanii* superclade. Because of limited sequence diversity and phylogenetic resolution of the 16S rRNA gene within the genus *Bradyrhizobium* [27,38,39], we refined the 16S rRNA phylogeny using concatenated gene sequences of housekeeping genes *gyrB-recA*, as this strategy has been adopted elsewhere [28]. This analysis showed that some cowpea-derived strains—particularly BR 12537 and BR 12538—belong to the same phylogenetic group as strain BR 3262 and notably share identical *nodC* sequences with it. Strain BR 3262 is a currently recommended inoculant for cowpea cultivation and was isolated from the same region and during the same period as the cowpea-derived strains in this study [40], suggesting it is a widely distributed genotype in the region.

Additionally, a distinct group of four strains (BR 10925, BR 10926, BR 10756 and BR 10760) obtained using cowpea as trap host, isolated from a pasture area, exhibited clear phylogenetic divergence from all *Bradyrhizobium* type strains based on analyses of the 16S rRNA gene, *gyrB-recA* and *nodC* (Figure 1, Figure 2 and Figure 3). This divergence was further corroborated by ANI and dDDH data, which confirmed the uniqueness of this clade. Although the closest related type strain was *B. cenepequi* CNPSo 4026^T^, the ANI and dDDH values suggest that these pasture-derived strains likely represent a novel species (Figure 4; Table 2).

Except for BR 13996, BR 13997 and BR 13999, the soybean-derived strains were distributed over two phylogenetic groups. One of these (G2, *gyrB-recA* Figure 2) harbors six strains similar to *B. elkanii* inoculant strains, particularly SEMIA 5019 and SEMIA 587, as evidenced by identical sequences for 16S rRNA, *gyrB-recA*, and *nodC* (Figure 1, Figure 2 and Figure 3). Also, among these six strains, only BR 13970 was isolated from plots that had not previously received soybean inoculants (Table 2). This suggests that these strains have originated from inoculation or eventually transmitted through the seed in the case of the BR 13970 [16].

The second soybean-associated group (G5, *gyrB-recA* Figure 2) joined strains from both inoculated and non-inoculated plots. Although their 16S rRNA sequences were closely related to multiple type strains, they formed a distinct monophyletic group based on *gyrB-recA* and *nodC* genes. ANI and dDDH analyses revealed that BR 13971, a representative of this group, indeed belongs to *B. elkanii* species with values exceeding 97% (ANI) and 83% (dDDH), respectively [26]. This group may represent a novel symbiovar capable of nodulating both soybean and cowpea, warranting further investigation [35]. It is important to note that the occurrence of soybean-nodulating strains in soils that have not received inoculants is rare in Brazil [15,37]. This suggests that such strains may have originated from previous inoculations and subsequently adapted to the local soil environment, potentially through horizontal gene transfer (HGT) of symbiotic genes [15]. The occurrence of HGT events among *Bradyrhizobium* strains under soybean cultivation has already been documented [8,15,41,42]. It has also been demonstrated that strains used as soybean inoculants can adapt to tropical soil conditions, persist, and become established in the soil via residues from inoculated soybean crops, although their population levels often remain low and may fluctuate depending on environmental conditions [15].

The *gyrB-recA* G3 comprised strains isolated from both cowpea and soybean. However, they were distributed into distinct groups in the *nodC* phylogeny (II and III), with each clade containing strains from both hosts (Figure 2, Figure 3, Table 1). Interestingly, while the soybean-derived strains were capable of nodulating cowpea, the reverse was not the case, that is, cowpea-derived strains failed to nodulate soybean. This pattern highlights the complexity of host–strain interactions within this group. The *gyrB-recA* phylogenetic analysis indicated that these seven strains share high sequence similarity with *B. brasilense* UFLA03-321^T^ and *B. australafricanum* CNPSo 4015^T^, but also with strain BR 3262, which has been previously identified as *B. pachyrhizi* and the *B. pachyrhizi* PAC 48^T^ (Figure 2). These findings suggest a high degree of genomic relatedness among strains from these four species, which also includes the *gyrB-recA* G3 strains examined in this study.

Whole-genome analyses of strain BR 10750 confirmed its high similarity to both *B. australafricanum* CNPSo 4015^T^ and *B. brasilense* UFLA03-321^T^, as indicated by ANI values of 95.1% and 98.8%, respectively (Figure 4; Table 1). A similar pattern was observed for the other strains within group G3 of the *gyrB–recA* phylogeny, particularly BR 13996 and BR 13998, which exhibited ANI values above 96.4% with *B. brasilense* UFLA03-321^T^.

Regarding dDDH, the genomes of these three strains showed values around or slightly above 70%, suggesting that BR 10750, BR 13996, and BR 13998 could be accommodated within either *B. brasilense* or *B. australafricanum* (Table 2). The proposed thresholds for species delineation are approximately 95–96% for ANI and 70% for dDDH [43,44].

It is also noteworthy that *B. brasilense* UFLA03-321^T^ and *B. australafricanum* CNPSo 4015^T^ themselves share an ANI of 96.3% and a dDDH value of 70%, both over the boundary for species definition. These findings indicate that a more comprehensive investigation, including additional strains and genome sequences, may be required to clarify the taxonomic relationships between the strains of this group.

In terms of nodulation ability and nitrogen fixation capacity, there does not appear to be a clear relationship between phylogeny based on different genes and the observed values. In the case of soybean, strain BR 13956 contributed significantly, at a level comparable to the reference strain SEMIA 5079, both in terms of nodulation and biomass production (Table 3). As previously indicated, this strain likely originated from inoculants containing strains SEMIA 587 or SEMIA 5019 used in the past. In contrast, for cowpea, strain BR 10926, representing a still-unknown taxonomic group, showed superior nodule biomass and greater contribution to shoot dry matter production than strain BR 3262, which is widely recognized as efficient for this crop [23]. This indicates that there are potentially superior strains than those currently recommended and highlights the need for more detailed field studies.

## 4. Materials and Methods

### 4.1. Bacterial Strains

Fourteen *Bradyrhizobium* strains were isolated from cowpea plants in the late 1990s, and twenty strains were obtained in 2018 from nodules of soybean plants. The bacteria isolated from cowpea were obtained by cultivating trap plants in soil collected from the Embrapa Agrobiologia experimental field station, located in Seropédica, Rio de Janeiro [40]. The soil had been collected from an area with a forest remnant, in a pasture area with manly *Brachiaria* spp. and an area cultivated annually with different crops (Table 1). The soybean strains originated from plants grown under field conditions, with area inoculated with strains recommended for soybean and non-inoculated (Table 1) [45]. Briefly, nodules collected from the plants were surface-sterilized (70% ethanol for 30 s, followed by 5% sodium hypochlorite for 3 min and ten successive rinses with sterile distilled water), then macerated, and the nodular contents streaked onto YMA culture medium. Successive subcultures were performed, when necessary, until pure cultures were obtained.

### 4.2. DNA Extraction, PCR, Sequencing Phylogenetic Analysis

Bacterial strains were cultivated in YM medium (4 days; 28 °C; 150 rpm), cultures were centrifuged and cell pellets were used for DNA extraction using the Bacterial Genomic DNA Isolation Kit (Wizard; Promega, Madison, WI, USA) with a few modifications [33]. The PCR for 16S rRNA, *recA*, *gyrB* a, nd *nodC* genes were performed as described following [33]. Amplicons were sequenced by Sanger technology, being forward and reverse reads of the 16S rRNA, *recA*, *gyrB* and *nodC* gene fragments processed using the Bionumerics package v. 7.0 (Applied Maths, Sint-Martens-Latem, Belgium). Initially, the sequences were compared to those deposited in the NCBI Nucleotide Database (https://www.ncbi.nlm.nih.gov/nucleotide, accessed on 8 August 2025) using the BLASTn tool. In the case of 16S rRNA, additional comparisons were performed using the EzBioCloud database (https://www.ezbiocloud.net/, accessed on 11 August 2025). Multiple sequence alignments and phylogenetic reconstructions using the Maximum Likelihood (ML) method were conducted with MEGA version X [46]. Distance matrices were calculated using the Kimura two-parameter substitution model [47], and the robustness of tree nodes was assessed with bootstrap analysis with 500 replicates. Default software parameters were used for all analysis. Tree visualization and editing were performed using the iTol package [48]. Sequences of type strains used for alignment and phylogenetic analysis were retrieved from GenBank (https://www.ncbi.nlm.nih.gov/genbank/, accessed on 14 August 2025). The NCBI ac. n°. are listed in Table S2.

### 4.3. Genome Sequencing, Assembly, Annotation, ANI and dDDH

For genome sequencing, libraries were constructed following the native barcoding genomic DNA protocol recommended by the manufacturer (Oxford Nanopore Technologies) (Oxford, UK; https://nanoporetech.com), after which sequencing was performed. The libraries were loaded onto a MinION flow cell model FLO-MIN 106 (version R10.3) and sequencing was monitored using the MinKNOW program (Oxford Nanopore Technologies). Base calling was performed using the Guppy program (Oxford Nanopore Technologies), data demultiplexing was performed using the Demultiplex program (version 1.2.1, available at https://github.com/jfjlaros/demultiplex), and sequencing data were evaluated using NanoPlot (version 1.24.0) [49]. Preprocessing next-generation sequencing (NGS) data was performed by Trimmomatic to deal with adapter removal and quality trimming [50]. Genomic sequences were assembled using the Flye program [51]. The evaluation of the assembled genomes was conducted using QUAST [52] and FASTQC [53]. Automatic genome annotation was performed with RASTtk [54]. The genome sequences were deposited in the GenBank RefSeq database for automatic annotation.

ANIclustermap [55] was employed to analyze the ANI among microbial genomes. The program calculates ANI values using either FastANI or Skani and visualizes the results as a clustermap. Genomic data in FASTA format was provided as input, and clustering was performed using the UPGMA method from SciPy. The clustermap was generated using Seaborn v0.13.2 (https://seaborn.pydata.org), with color-coded annotations representing ANI values. Parameters such as figure dimensions and dendrogram ratios were adjusted to optimize visualization. The analysis was conducted on Python 3.8 or later, with dependencies installed via Bioconda or PyPI.

To estimate dDDH, the bacterial genomes were analyzed using the Type Strain Genome Server (TYGS; https://tygs.dsmz.de, accessed on 12 September 2025) [56]. The genome sequences were compared with those of type strains of *Bradyrhizobium* species. The dDDH values were calculated using the GGDC (Genome-to-Genome Distance Calculator) integrated within TYGS, applying formula d_4_ *d4* (a.k.a. GGDC formula 2), which computes intergenomic relatedness independently of genome length and yields results highly consistent with empirical DNA–DNA hybridization data.

### 4.4. Plant Inoculation Tests

Three experiments were conducted with cowpea (cv. BRS Guariba) or soybean (cv. BRS 5980IPRO), under axenic conditions in Leonard jars using an autoclaved mixture of sand and vermiculite (1:1) [57], using Norris’s nutrient solution and watering as needed [58]. The nutrient solution in the lower compartment of the jars was discarded weekly and replaced with 300 mL of sterilized nutrient solution. A randomized complete block design with three replicates was used. Seed surface sterilization was performed with immersion in ethanol (70%) for 1 min, followed by hydrogen peroxide (35%) for 3 min and finally 10 successive washes with sterilized distilled water.

The first experiment aimed to confirm the ability of the strains to nodulate both hosts. Three soybean and three cowpea seeds were sown together in each Leonard jar. Immediately after seedling emergence, two plants of each host were maintained per jar, and inoculation was performed. All 34 strains were grown on YMA medium for 3–4 days until reaching an optical density close to 1.0 (about 10^9^ cells mL^−1^) and 1 mL of the grown culture was inoculated onto each seedling of each host. Sampling was performed 35 days after plant emergence by evaluating nodulation and the shoot dry biomass (dried in an oven at 65 °C for 24 h).

The second experiment was conducted with Leonard jars, similarly to the first experiment, cultivated with cowpea to evaluate the performance of strains BR 10926, BR 10750, BR 13971, BR 13956, and the positive control *B. pachyrhizi* BR 3262. In this experiment, the *B. pachyrhizi* BR 3262 (positive control) was also included, along with a nitrogen-fertilized treatment (50 mg N per week as ammonium nitrate) and one absolute control without nitrogen and without inoculation. In the third experiment, the strains BR 13956 and BR 13971 and the positive control *B. japonicum* SEMIA 5079 were inoculated onto soybean alongside nitrogen-fertilized and absolute control treatments, similarly to the soybean experiment. Inoculation was performed as mentioned before.

The second and third experiments were conducted for 40 days, after which nodulation and shoot dry biomass (dried in an oven at 65 °C for 24 h) were evaluated. Data were analyzed by ANOVA and means compared using Tukey’s test (5%).

## 5. Conclusions

This study provides a comprehensive phylogenetic and genomic assessment of 34 *Bradyrhizobium* strains isolated from cowpea and soybean nodules in Brazil, revealing both conserved relationships and substantial diversification within the genus. Multilocus phylogenetic analyses (16S rRNA, *gyrB*, *recA*, and *nodC*), supported by whole-genome comparisons using ANI and dDDH, demonstrated that while many strains cluster within the *B. elkanii* superclade, several form distinct and deeply divergent lineages, indicating the presence of previously unrecognized taxa. These findings highlight Brazilian *Bradyrhizobium* populations as reservoirs of unexplored genetic diversity and reinforce predictions of a high number of yet undescribed species within the genus. Symbiotic evaluations showed marked variability in nodulation ability and efficiency, with one group of cowpea-derived strains exhibiting high symbiotic performance despite low genomic similarity to known type strains, suggesting strong potential for novel inoculant development. Particularly for cowpea, strains BR 10926 and BR 10750 demonstrated higher symbiotic efficiency than the strains currently recommended for this crop. Conversely, some soybean-derived strains were genetically indistinguishable from commercial inoculants, indicating either persistence in the soil or re-isolation from previously inoculated fields. Cross-inoculation assays further revealed asymmetrical host compatibility: soybean-derived strains effectively nodulated cowpea, whereas cowpea-derived strains did not nodulate soybean. Notably, strain BR 13971 displayed certain efficient nodulation on both hosts, suggesting the existence of a broad-host-range symbiovar with promising agronomic potential. Collectively, these results expand our understanding of *Bradyrhizobium* diversity in tropical soils and underscore the importance of integrating genomic, physiological, and agronomic approaches to identify elite strains for sustainable agricultural applications.

## Figures and Tables

**Table 1:** Hosts, locals of collection, genotypic groups of the *Bradyrhizobium* strains based on 16S rRNA, *recA-gyrB* and *nodC*, and cross-inoculation response (NN—nodules number per plants, MN—nodules dry mass (mg plant^−1^), DM—plant dry matter (g plant^−1^).

Host	Local	Coord.	Strains	Groups			Cowpea			Soybean		
				16S rRNA	*recA-* *gyrB*	*nodC*	NN	MN	DM	NN	MN	DM
Cowpea	Pasture	22°45′10″ S 43°40′25″ W	BR 10926	1	G1	I	0.4	0.3	0.9	0.0	0.0	0.1
			BR 10925				0.7	0.7	0.7	0.0	0.0	0.1
			BR 10756				0.6	0.7	0.7	0.0	0.0	0.1
			BR 10760				0.4	0.3	0.3	0.0	0.0	0.1
			BR 10735	2	G3	II	0.7	0.5	0.6	0.0	0.0	0.1
			BR 10741				0.7	0.6	0.6	0.0	0.0	0.1
			BR 10750				0.4	0.3	0.9	0.0	0.0	0.1
			BR 12538			III	0.8	0.7	0.4	0.0	0.0	0.1
	Area cultivated	22°45′11″ S 43°40′26″ W	BR 12537		G3	III	0.7	0.5	0.5	0.0	0.0	0.1
	Forest	22°45′04″ S 43°40′18″ W	BR 10733		G4	VI	0.7	0.6	0.6	0.0	0.0	0.1
			BR 10738				0.7	0.6	0.6	0.0	0.0	0.1
			BR 10739				0.7	0.6	0.6	0.0	0.0	0.1
			BR 10740				0.4	0.2	0.1	0.0	0.0	0.1
			BR 10746				0.7	0.6	0.6	0.0	0.0	0.1
Soybean	Area with soybean inoculated	22°44′51″ S 43°40′15″ W	BR 13916		G2	VI	0.2	0.3	0.1	0.2	0.2	0.2
			BR 13917				0.8	0.7	0.2	0.3	0.5	0.3
			BR 13918			V	0.3	0.2	0.2	0.8	0.9	1.0
			BR 13956				0.3	0.2	0.5	1.0	0.7	1.0
			BR 13970				0.3	0.2	0.5	0.8	0.9	0.8
			BR 13998		G3	II	0.5	0.4	0.4	0.2	0.5	0.4
			BR 13996			III	0.3	0.3	0.2	0.1	0.1	0.1
			BR 13997				0.3	0.2	0.5	0.1	0.1	0.4
			BR 13971		G5	IV	0.9	1.0	1.0	0.3	0.2	0.3
			BR 13973				1.0	0.9	0.5	0.4	0.5	0.3
			BR 13986		G2	V	0.9	0.6	0.4	0.4	0.4	0.4
			BR 13974		G5	IV	0.9	0.2	0.4	0.4	0.1	0.4
	Area with soybean uninoculated	22°44′53″ S 43°40′13″ W	BR 13891				0.5	0.7	0.5	0.3	0.2	0.3
			BR 13892				0.8	0.4	0.3	0.3	0.5	0.2
			BR 13893				0.5	0.7	0.5	0.3	0.2	0.4
			BR 13894				0.6	0.7	0.5	0.3	0.2	0.4
			BR 13895				0.6	0.4	0.3	0.4	0.8	0.3
			BR 13984				0.9	0.9	0.7	0.3	0.3	0.2
			BR 13985				0.3	0.2	0.4	0.1	0.1	0.5
			BR 14157				0.4	0.2	0.4	0.5	0.5	0.6
			Control				0.0	0.0	0.1	0.0	0.0	0.1
			Control				0.0	0.0	0.1	0.0	0.0	0.1
				Host	NN	MN	DM		1 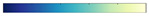 0			
				Soybean	54.5	215.0	2.3	Max	Normalized value (0–1)			
				Cowpea	60	201.5	2.5					
				Soybean	0	0	0.3	Min.				
				Cowpea	0	0	0.3					

**Table 2:** Average Nucleotide Identity (ANI) and digital DNA–DNA Hybridization (dDDH) among different *Bradyrhizobium* strains.

Strains	BR 13971	USDA 76^T^	BR 10750	BR 13996	BR 13998	CNPSo 4015^T^	UFLA 03-321^T^	BR 10926	CNPSo 4026^T^	PAC 48^T^
ANI (%)										
BR 13971	-	98.1	94.7	94.7	94.8	94.8	94.8	94.7	83.3	83.2
*B. elkanii* USDA 76^T^	98.1	-	94.4	94.6	94.6	94.7	94.7	94.6	83.3	83.2
BR 10750	94.7	94.4	-	98.0	99.8	96.2	99.0	95.2	83.4	83.2
BR 13996	94.7	94.6	98.0	-	98.1	96.9	98.1	95.4	83.4	83.2
BR 13998	94.8	94.6	99.8	98.1	-	96.4	99.2	95.4	83.4	83.2
*B. australafricanum* CNPSo 4015^T^	94.8	94.7	96.2	96.9	96.4	-	96.3	95.4	83.3	83.3
*B. brasilense* UFLA03-321^T^	94.8	94.7	99.0	98.1	99.2	96.3	-	95.4	83.5	83.3
*B. pachyrhizi* PAC 48^T^	94.7	94.6	95.2	95.4	95.4	95.4	95.4	-	83.3	83.1
BR 10926	83.3	83.3	83.4	83.4	83.4	83.3	83.5	83.3	-	89.5
*B. cenepequi* CNPSo 4026^T^	83.2	83.2	83.2	83.2	83.2	83.3	83.3	83.1	89.5	-
dDDH (%)										
BR 13971	-	83.6	59.3	58.6	59.7	58.7	61.9	25.5	25.4	57.0
*B. elkanii* USDA 76^T^	83.6	-	58.6	58.6	59.0	58.8	61.8	25.6	25.5	57.4
BR 10750	59.3	58.6	-	84.6	99.0	70.7	68.9	25.6	25.3	62.6
BR 13996	58.6	58.6	84.6	-	85.5	73.8	94.2	25.5	25.4	63.9
BR 13998	59.7	59.0	99.0	85.5	-	71.8	85.7	25.6	25.3	63.0
*B. australafricanum* CNPSo 4015^T^	58.7	58.8	70.7	73.8	71.8	-	70.0	25.5	25.4	62.2
*B. brasilense* UFLA03-321^T^	61.9	61.8	68.9	94.2	85.7	70.0	-	25.5	25.4	62.8
*B. pachyrhizi* PAC 48^T^	57.0	57.4	62.6	63.9	63.0	62.2	62.8	25.4	25.3	-
BR 10926	25.5	25.6	25.6	25.5	25.6	25.5	25.5	-	39.5	25.4
*B. cenepequi* CNPSo 4026^T^	25.4	25.5	25.3	25.4	25.3	25.4	25.4	39.5	-	25.3

The values highlighted in gray indicate those that are equal to or above the threshold for species delineation in each method.

**Table 3:** Nodule number, nodule dry mass, and plant dry matter of cowpea and soybean plants inoculated with different *Bradyrhizobium* strains *.

Strain	Number of Nodules		Nodules Dry Mass (mg plant^−1^)		Plant Dry Matter (g plant^−1^)	
Cowpea						
BR 10926	130.7	A	220.3	A	4.3	A
BR 10750	133.2	A	245.7	A	4.2	A
Nitrogen	0.0	E	0.0	D	3.9	A
BR 13971	84.0	B	92.0	C	3.6	AB
BR 3262	106.2	A	83.7	C	3.6	B
BR 13956	54.0	D	41.8	CD	2.4	C
Control	0.0	E	0.0	D	1.1	D
C.V. (%)	19.6		31.82		9.37	
Soybean						
BR 13956	117.7	A	294.7	A	4.3	A
BR 13971	96.0	A	125.0	B	2.8	B
SEMIA 5079	91.0	A	336.3	A	4.0	AB
Control	0.0	B	0.0	C	1.3	C
Nitrogen	0.0	B	0.0	C	5.4	A
C.V. (%)	27.2		26.76		17.2	

* Means followed by the same letters in the columns and for each host do not differ significantly according to Tukey’s test at the 5% significance level. Control refers to treatments without inoculation or nitrogen fertilization; Nitrogen refers to 50 mg N per week. C.V. (%): Coefficient of variation.

## Data Availability

The gene and genome sequence data are available in NCBI, and that other information could be provided on request.

## Supplementary Materials

The following supporting information can be downloaded at https://www.mdpi.com/article/10.3390/plants14243857/s1: Figure S1: Maximum likelihood phylogeny of partial 16S rRNA sequences of Bradyrhizobium strains. Bootstrap values over 50% based on 500 replicates are shown.; Table S1: Technical information from genomes assembly; Table S2: NCBI ac. n°. of the Bradyrhizobium strains and other representative type strain of the genus.

## Abbreviations

The following abbreviations are used in this manuscript:

ANI	Average Nucleotide Identity
dDDH	digital DNA–DNA Hybridization
*recA*	Recombinase A gene
*gyrB*	DNA gyrase subunit B gene
*nodC*	Nodulation protein C gene
