# Supplementary material for: Genetic Diversity and Nodulation Potential of Bradyrhizobium Strains in Cowpea and Soybean
Source: Plants (Basel). 2025 Dec 18;14(24):3857. doi: 10.3390/plants14243857 (PMC12737117; doi:10.3390/plants14243857)
Supplement: Supplementary file 1 [file plants-14-03857-s001.zip › plants-3977047-supplementary.pdf]

Table S1. Technical information from genomes assembly.

Features	BR 13971	BR 10926	BR 10750	BR 13996	BR 13998
Genome size	8.7 Mb	10 Mb	8.9 Mb	8.5	8.4
Number of contigs	11	6	1	3	136
Contig N50	3.2 Mb	9.4 Mb	8.9	8.5	8.4
GC percent	64	62.5	63.8	63.8	63.7
Genome coverage	13x	14x	18x	17x	11x

Table S2. NCBI ac. n°. of the Bradyrhizobium strains and other representative type strain of the genus.

Species/Strain	16S rRNA	*gyrB*	*recA*	*nodC*	genome
BR 10733	PV588884	PX246236	PX206272	PX206286	-
BR 10735	PV588885	PX246242	PX206278	PX206292	-
BR 10738	PV588886	PX246237	PX206273	PX206287	-
BR 10739	PV588887	PX246238	PX206275	PX206288	-
BR 10740	PV588888	PX246235	PX206271	PX206285	-
BR 10741	PV588889	PX246241	PX206277	PX206291	-
BR 10746	PV588890	PX246239	PX206274	PX206289	-
BR 10750	PV588891	PX246248	PX206284	PX206298	PRJNA1258923
BR 10756	PV588892	PX246246	PX206282	PX206296	-
BR 10760	PV588893	PX246245	PX206281	PX206295	-
BR 10925	PV588894	PX246243	PX206279	PX206293	-
BR 10926	PV588895	PX246244	PX206280	PX206294	GCA_052047415.1
BR 12537	PX244310	PX246240	PX206276	PX206290	-
BR 12538	PV588897	PX246247	PX206283	PX206297	-
BR 13891	PV588899	PX246215	PX206251	PX206299	-
BR 13892	PV588900	PX246216	PX206252	PX206300	-
BR 13893	PV588901	PX246217	PX206253	PX206301	-
BR 13894	PV588902	PX246218	PX206254	PX206302	-
BR 13895	PV588903	PX246219	PX206255	PX206303	-
BR 13916	PV588904	PX246226	PX206262	PX206310	-
BR 13917	PV588905	PX246227	PX206263	PX206311	-
BR 13918	PV588906	PX246228	PX206264	PX206312	-
BR 13956	PV588907	PX246229	PX206265	PX206313	-
BR 13970	PV588908	PX246230	PX206266	PX206314	-
BR 13971	PV588909	PX246220	PX206256	PX206304	GCA_052050015.1
BR 13973	PV588910	PX246221	PX206257	PX206305	-
BR 13974	PV588911	PX246222	PX206258	PX206306	-
BR 13984	PV588912	PX246223	PX206259	PX206307	-
BR 13985	PV588913	PX246224	PX206260	PX206308	-
BR 13986	PV588914	PX246231	PX206267	PX206315	-
BR 13996	PV588915	PX246232	PX206268	PX206316	SAMN52657657
BR 13997	PV588916	PX246233	PX206269	PX206317	-
BR 13998	PV588917	PX246234	PX206270	PX206318	SAMN52900451
BR 14157	PX244309	PX246225	PX206261	PX206309	-
B. acaciae 10BB^T	LR877298	genome	genome	genome	GCF_020889785.1
B. aeschynomenes 83002^T	KU991833	genome	genome	genome	GCF_013178945.1
B. agreste CNPSo 4010^T	KP768779	MK860852	MK863432	MK893236	-
B. algeriense RST89^T	LR877293	genome	genome	FJ348666	GCF_036924595.1

B. altum Pear77^T	MK676065	genome	genome	genome	GCF_020889705.1
B. amazonense INPA03-11B^T	MK676054	genome	genome	genome	GCF_040446015.1
B. americanum CMVU44^T	MK676067	-	KC247141	KC247130	-
B. amphicarpaeae 39S1MB^T	KP768818	KP768721	KF615002	-	-
B. arachidis CCBAU 51107^T	AB681928	JX437675	HM107233	KF962705	-
B. archetypum CNPSo 4013^T	KF311068	MK860868	MK863448	MK893251	-
B. australafricanum CNPSo 4015^T	KY349447	genome	MK863437	MK893241	GCF_020329495.1
B. australiense CNPSo 4014^T	KU230298	MK860870	MK863450	MK893253	-
B. barranii 144S4^T	AJ558025	genome	genome	KF615639	GCF_017565645.3
B. betae NBRC 103048^T	MK676055	FM253217	AB353734	-	-
B. brasilense UFLA03 321^T	KF927049	genome	genome	KT793173	GCA_001969825.1
B. cajani AMBPC1010^T	KC247115	genome	KY349440	KY349444	GCF_024199785.1
B. campsiandrae INPA 394B^T	Z94812	KT793134	KT793146	-	-
B. canariense BTA-1^T	KP768789	FM253220	FM253177	AJ560653	-
B. cenepequi CNPSo 4026^T	EU561065	MK860859	MK863439	MK893243	-
B. centrolobii BR 10245^T	KJ184551	KX528004	KX527954	KX527941	-
B. centrosematis A9^T	AF338176	genome	KC247145	KC247134	GCF_024809395.1
B. commune BDV5040^T	D13430	genome	genome	-	GCF_015624505.1
B. cosmicum 58S1^T	MK676046	KP768731	KF615104	-	-
B. cytisi CTAW11^T	AB509378	KF532653	GU001575	EU597844	-
B. daqingense CCBAU 15774^T	AY904773	JX437669	HQ231270	HQ231326	-
B. denitrificans IFAM 1005^T	KF114645	FM253239	FM253196	-	-
B. diazoefficiens USDA 110^T	KX683400	genome	genome	genome	GCF_000011365.1
B. diversitatis CNPSo 4019^T	KR779520	MK860850	MK863430	MK893234	-
B. elkanii NBRC 14791^T	MK676047	genome	AY591568	AB354631	GCF_023278185.1
B. embrapense SEMIA 6208^T	MT760081	HQ634891	HQ634899	KP234521	GCF_001189235.2
B. erythrophlei CGMCC 1.13002^T	KC508877	KF114717	KF114669	-	GCF_042430365.1
B. ferriligni CCBAU 51502^T	KC508852	KJ818102	KJ818112	KJ818109	-
B. forestalis INPA 54B^T	MK676061	genome	genome	KT793177	GCF_002795245.1
B. frederickii CNPSo 3426^T	MK458611	MK682721	MK682710	MK682743	-
B. ganzhouense RITF806^T	HQ231463	KP420022	JX277144	JX292035	-
B. glycinis CNPSo 4016^T	KF896156	MK860851	MK863431	MK893235	-
B. guangdongense CGMCC 1.15034^T	KF927043	KC509072	KC509269	genome	GCF_014640515.1
B. guangxiense CCBAU 53363^T	AB681854	KC509082	KC509279	genome	GCF_004114915.1
B. guangzhouense CCBAU 51670^T	KX396570	KC509057	KC509254	genome	GCF_004114445.1
B. hereditatis CNPSo 4025^T	X87272	MK860864	MK863444	MK893247	-
B. hipponense aSej3^T	AY624134	genome	genome	genome	GCF_008123965.1
B. huanghuaihaiense CCBAU 23303^T	KP899562	JX437672	HQ231595	KF472814	-
B. icense LMTR13^T	GU433448	KF896201	JX943615	KF896159	-
B. ingae BR 10250^T	AF208513	KF927079	KF927061	KF927054	-
B. iriomotense NBRC 102520^T	LNCU01000022	AB300997	AB300996	AB301000	GCF_030160715.1
B. ivorense CI-1B^T	HQ641226	MH756161	MK376330	genome	GCF_020889745.1
B. japonicum DSM 30131^T	FJ025102	AB070586	AM182158	AP012206	-
B. jicamae PAC 68^T	MK676062	HQ873309	HM047133	AB573869	-
B. kavangense 14-3^T	KX661401	KX661397	KM378399	KT033402	-
B. lablabi CCBAU 23086^T	KC508872	JX437670	GU433522	GU433565	-
B. liaoningense 2281^T	KF927051	FM253223	FM253180	GU263466	-

B. lupini USDA3051ᵀ	MK673807	genome	KM114866	KM114864	GCF_040939785.1
B. macuxiense BR 10303ᵀ	AB542368	KX528008	KX527958	KX527945	GCF_001542415.1
B. manausense BR 3351ᵀ	D78366	KF786000	KF785992	KF786002	-
B. mercantei SEMIA 6399ᵀ	CP088156	KX690623	KX690615	-	GCF_001982635.1
B. murdochi CNPSo 4020ᵀ	LR877297	MK860865	MK863445	MK893248	-
B. namibiense 5-10ᵀ	JN186270	KX661393	KM378377	KX661399	-
B. nanningense CCBAU 53390ᵀ	AY624135	KC509077	KC509274	genome	GCF_004114535.1
B. neotropicale BR 10247ᵀ	AY923031	KJ661707	KJ661714	KJ661727	-
B. niftali CNPSo 3448ᵀ	MZ486441	MK675794	MK675797	genome	GCF_004571025.1
B. nitroreducens TSA1ᵀ	KP768782	genome	genome	-	GCF_002776695.1
B. oligotrophicum JCM 1494ᵀ	KC247085	KF962697	JQ619231	-	-
B. ontarionense A19ᵀ	EU561074	genome	genome	-	GCF_021088345.1
B. oropedii Pear76ᵀ	MF593081	LN650208	-	-	GCF_020889685.1
B. ottawaense OO99ᵀ	MZ315001	HQ873179	HQ587287	HQ587980	-
B. pachyrhizi PAC 48ᵀ	MZ315000	HQ873310	HM047130	AB573868	GCA_001189245.1
B. paxllaeri LMTR 21ᵀ	NZ_JAGKJJ 010000023	KF896195	JX943617	KF896160	-
B. quebecense 66S1MBᵀ	KP768787	KP768724	KF615025	KF615618	GCA_013373795.3
B. retamae Ro19ᵀ	FMAI01000 010	KF896204	KF962711	KC247112	-
B. rifense CTAW71ᵀ	KU724142	KC569466	GU001585	EU597853	-
B. ripae WR 4ᵀ	KP308152	MF593094	MF593090	MF593106	-
B. sacchari P9-20ᵀ	KP768783	genome	KX065095	KF196792	GCF_002068095.1
B. sediminis S2-20-1ᵀ	AY904753	genome	MZ496283	-	GCF_018736085.1
B. semiaridum CNPSo 4028ᵀ	NZ_VKHP 01000252	MK860860	MK863440	genome	GCF_020329505.1
B. septentrionale 1S1ᵀ	JX514883	KP768729	KF615049	KF615620	GCF_011516645.4
B. shewense ERR 11ᵀ	KP899563	genome	genome	genome	GCF_900094605.1
B. stylosanthis BR 446ᵀ	FJ025107	KU724151	KU724163	KU724160	-
B. subterraneum 58 2-1ᵀ	CP089391	KX661396	KM378397	MH182918	-
B. symbiodeficiens 85S1MBᵀ	AF193818	KP768725	KF615036	-	-
B. tropiciagri SEMIA 6148ᵀ	KC508861	HQ634890	FJ391168	KP234520	GCF_001189845.1
B. uaiense UFLA03 164ᵀ	ON307082	KT793133	KT793144	genome	GCF_010811875.1
B. valentinum LmjM3ᵀ	JASGSR010 000002	genome	JX518589	JX514897	GCF_001440405.1
B. vignae 7-2ᵀ	NSJY01000 003	genome	KM378374	KT362339	GCF_900324035.1
B. viridifuturi SEMIA 690ᵀ	ADOU0200 0007	KR149134	KR149140	genome	GCF_001238275.1
B. xenonodulans 14ABᵀ	AF237422	genome	LR899498	genome	GCF_027594865.1
B. yuanmingense CCBAU 10071ᵀ	AF234890	FM253226	FM253183	AB354633	-
B. zhanjiangense CCBAU 51778ᵀ	AF234888	KC509066	KC509263	genome	GCF_004114435.1
B. zhengyangense WYCCWR 13023ᵀ	FJ546419	genome	MW168373	genome	GCF_022012435.1
B. prioriisuperbiae BL16Aᵀ	MK559111	genome	genome	genome	GCF_032397745.1
B. roseum S12-14-2ᵀ	MK676048	genome	genome	genome	GCF_030413175.1
B. viridifuturi UFLA03-84	AY649430	genome	genome	genome	GCA_002289535.1
B. japonicum SEMIA 5079ᵀ	genome	genome	genome	genome	GCA_021229285.1
B. diazoefficiens SEMIA 5080ᵀ	genome	genome	genome	genome	GCA_000648595.2
B. elkanii Semia 5019	MK672937	genome	genome	genome	GCA_013392745.1
B. elkanii SEMIA 587ᵀ	JQ796661	genome	genome	genome	GCA_036936725.1
B. pachyrhizi BR3262ᵀ	KF113091	genome	genome	genome	GCA_001440015.1
B. yuanmingense BR 3267ᵀ	genome	genome	genome	genome	GCA_001439885.1

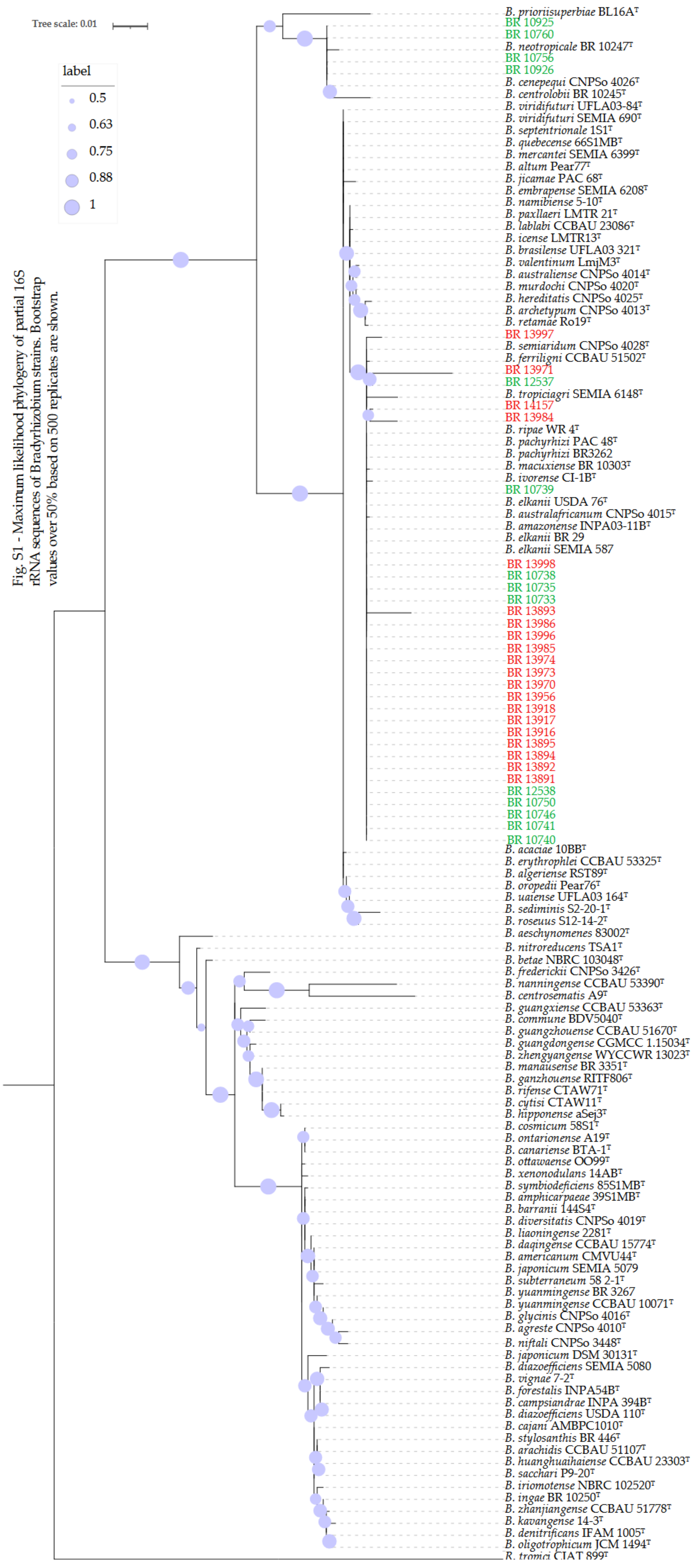

Fig. S1 - Maximum likelihood phylogeny of partial 16S rRNA sequences of Bradyrhizobium strains. Bootstrap values over 50% based on 500 replicates are shown.
